# The Evaluation of Ordinal Regression Model's Performance Through the Implementation of Multilayer Feed-Forward Neural Network: A Case Study of Hypertension

**DOI:** 10.7759/cureus.54387

**Published:** 2024-02-18

**Authors:** Mohamad N Adnan, Wan Muhamad Amir W Ahmad, Hazik B Shahzad, Faiza Awais, Nor Azlida Aleng, Nor F Noor, Mohamad Shafiq B Mohd Ibrahim, Noor Maizura M Noor

**Affiliations:** 1 School of Dental Sciences, Universiti Sains Malaysia, Kota Bharu, MYS; 2 Department of Community and Preventive Dentistry, Rashid Latif Dental College, Lahore, PAK; 3 Faculty of Computer Science and Mathematics, Universiti Malaysia Terengganu, Kuala Terengganu, MYS; 4 Faculty of Medicine, Universiti Sultan Zainal Abidin, Kuala Terengganu, MYS; 5 Faculty of Dentistry, International Islamic University Malaysia, Kuantan, MYS

**Keywords:** mlffnn, bootstrap, r-square, ordinal logistic regression, hypertension

## Abstract

Background

Hypertension, or high blood pressure, is a common medical condition that affects a significant portion of the global population. It is a major risk factor for cardiovascular diseases (CVD), stroke, and kidney disorders.

Objective

The objective of this study is to create and validate a model that combines bootstrapping, ordered logistic regression, and multilayer feed-forward neural networks (MLFFNN) to identify and analyze the factors associated with hypertension patients who also have dyslipidemia.

Material and methods

A total of 33 participants were enrolled from the Hospital Universiti Sains Malaysia (USM) for this study. In this study, advanced computational statistical modeling techniques were utilized to examine the relationship between hypertension status and several potential predictors. The RStudio (Posit, Boston, MA) software and syntax were implemented to establish the relationship between hypertension status and the predictors.

Results

The statistical analysis showed that the developed methodology demonstrates good model fitting through the value of predicted mean square error (MSE), mean absolute deviance (MAD), and accuracy. To evaluate model fitting, the data in this study was divided into distinct training and testing datasets. The findings revealed that the results strongly support the superior predictive capability of the hybrid model technique. In this case, five variables are considered: marital status, smoking status, systolic blood pressure, fasting blood sugar levels, and high-density lipoprotein levels. It is important to note that all of them affect the hazard ratio: marital status (β1, -17.12343343; p < 0.25), smoking status (β2, 1.86069121; p < 0.25), systolic blood pressure (β3, 0.05037332; p < 0.25), fasting blood sugar (β4, -0.53880322; p < 0.25), and high-density lipoprotein (β5, 5.38065556; p < 0.25).

Conclusion

This research aims to develop and extensively evaluate the hybrid approach. The statistical methods employed in this study using R language show that regression modeling surpasses R-squared values in predicting the mean square error. The study's conclusion provides strong evidence for the superiority of the hybrid model technique.

## Introduction

Hypertension, also known as high blood pressure, is a prevalent medical condition affecting a substantial portion of the global population [1]. It is a major risk factor for cardiovascular diseases (CVD), including heart attacks; kidney disease; and strokes, contributing to significant morbidity and mortality worldwide [2]. In line with this, a 34-year follow-up of the Framingham Heart Study cohort found that those with higher blood pressure quintiles had a greater risk of congestive heart failure than those in the lower quintile at the start of the study [3]. The American Heart Association (AHA) and the American College of Cardiology (ACC) offer recommendations for the management and treatment of cardiovascular diseases. These guidelines are based on the most current evidence available and aim to improve patient outcomes by providing healthcare professionals with clear, evidence-based recommendations for diagnosis, treatment, and prevention strategies. They define hypertension as persistently elevated blood pressure levels, with values exceeding 130/80 mmHg [4]. The prevalence of hypertension has been steadily increasing, driven by an aging population, sedentary lifestyles, unhealthy dietary patterns, and other environmental factors [5]. Epidemiological studies have revealed the widespread prevalence of hypertension. According to global estimates, approximately 1.13 billion individuals are affected by hypertension, accounting for 25% of the adult population [6]. The National Health and Nutrition Examination Survey (NHANES) shows that the rate of hypertension among US adults increased from 23.9% in 1988-1994 to 29.0% in 2007-2008. However, the National Health and Morbidity Survey (NHMS) reported that hypertension among Malaysian adults was 30.3% (rates increasing with age) [2].

The World Health Organization has stated that hypertension disproportionately affects populations in low- and middle-income countries with weak health systems [7]. Large-scale data from 52 countries showed that those with severely raised blood pressure have a 2.5-fold higher risk of myocardial infarction than those with normal blood pressure, regardless of ethnicity, sex, or smoking status [3]. Not only were people with clinical hypertension at risk, but also people with prehypertension had a 1.5 times greater chance of developing cardiovascular disease than people with normal blood pressure [8]. The burden of hypertension varies across countries and is influenced by various factors, including age, gender, ethnicity, socioeconomic status, and lifestyle choices [6]. The pathophysiology of hypertension involves intricate interactions between genetic, environmental, and lifestyle factors, leading to increased vascular resistance, altered fluid balance, and the dysregulation of the renin-angiotensin-aldosterone system (RAAS) [9]. The risk factors for hypertension encompass a wide range of factors, including age, family history, obesity, sedentary lifestyle, excessive sodium intake, alcohol consumption, and socioeconomic status [4]. Understanding these risk factors and their interplay is vital for developing targeted interventions [8,9].

Asian nations, particularly those with high levels of industrialization, are at risk from an epidemic of hypertension. Over 180 million people in China were thought to have hypertension in 2000, and it was predicted that this number would rise by about 100 million people by 2025 [10]. Despite the policies and task forces on hypertension that the government has put in place, the prevalence of hypertension in Malaysia has remained high over the past few decades and has not significantly decreased in the community [11]. According to recent population-based studies conducted in Malaysia, hypertension is disproportionately more prevalent in males, older populations, and people with lower household incomes [12]. By critically evaluating the latest research, we aim to provide healthcare professionals and policymakers with an updated overview of the potential benefits and limitations of lifestyle modifications in hypertension management. Such insights can help inform clinical practice, guideline development, and public health initiatives, ultimately contributing to more effective strategies for preventing and controlling hypertension.

## Materials and methods

Study design and data collection

This study utilizes an advanced computational statistical modeling methodology, specifically supervised machine learning, combining multilayer feed-forward neural network (MLFFNN) with ordinal regression to develop a novel approach tailored to achieve the objectives. The methodology involves randomly dividing the data into two groups, namely, the 70% testing and 30% training datasets. The developed methodology relies on various factors, such as the testing and training datasets, mean square error (MSE)-predicted values, and the accuracy of the mean absolute deviance (MAD). The significance level chosen was p < 0.25, aligning with the methodology outlined by Mickey and Greenland [13] in their research on regression, to discern variables deemed significant [14]. The data for this study was obtained from the Hospital Universiti Sains Malaysia (USM). The confidentiality of the patient's information and medical status was ensured. Hypertension data included three ordinal categories: normal blood pressure, borderline high blood pressure, and high blood pressure. Categorizing blood pressure into ordinal categories is preferred as it mirrors clinical practice and enables clearer comparisons and the identification of trends. Table 1 provides a summary of the data descriptions for the research variables. Ethical approval for the study was obtained from the Universiti Sains Malaysia Research Ethics and Human Research Committee (USM/JEPeM/16050184), ensuring the protection of patient privacy and medical information.

**Table 1:** Data description of the selected blood profile

Variable	Code	Type	Description
Hypertension	Y	Categorical	Hypertension Status: 0 = Normal, 1 = Borderline, and 2 = High
Marital	X_1_	Categorical	Marital Status: 0 = Single and 1 = Married
Smoking Status	X_2_	Categorical	Smoking Status: 1 = Never, 2 = Formal, and 3 = Current
Sys	X_3_	Numeric	Systolic Reading
Fbs	X_4_	Numeric	Fasting Blood Sugar
Hdl	X_5_	Numeric	High-Density Lipoprotein

Modeling of computational biometry

In this study, ordered logistic regression was used to analyze the data using the RStudio program (Posit, Boston, MA). R is an open-source programming language, and in this study, R version 4.3.1 (Beagle Scouts) (R Foundation for Statistical Computing, Vienna, Austria) was used. The advanced methodology employed in this study is a combination model that incorporates various approaches, including bootstrap, multilayer feed-forward neural networks, and ordinal regression. The technique utilized in this study involves dividing the data into two distinct phases, similar to training and testing data. Phase I is dedicated to modeling, while Phase II is focused on validation. Specifically, the training data is used for developing the multilayer neural networks, while the testing data is used for validation purposes. In Phase I, a set of multilayer neural networks is fitted, and in Phase II, ordinal regression models are employed to examine the underlying relationship between hypertension and the selected explanatory variables.

Bootstrap

The bootstrap method begins by selecting a random sample from a population, and sample statistics are calculated based on this sample. Subsequently, a "pseudo-population" is created by generating multiple subsamples with replacements, where each subsample is a replica of the original sample. With replacement sampling, the random selection process can result in samples that differ from the original sample. As the bootstrap process progresses, statistics are computed for each sample drawn with replacement [15,16]. In this study, the ordinal logistic regression (OLR) is fitted using the R software.

Ordinal Regression

Ordinal regression is a useful technique for modeling a categorical dependent variable that has more than two categories of one or more independent variables. Specifically, ordinal logistic regression (OLR) is employed when the response variable exhibits a natural rank or order and contains more than two categories. It is important for the dependent variable to have an ordinal scale in order to use ordinal logistic regression. In the context of this research, the hypertension reading, which has a ratio measurement scale, needs to be transformed into a three-scaled ordinal variable. After converting hypertension readings to an ordinal scale, ordinal regression will be performed. The value of the regression parameter will be estimated using the maximum likelihood method. The model for ordinal regression is given. The dependent variable, however, is categorized; therefore, we must use the following: $C_x (x)=ln((\sum pr(event))/(1-\sum pr(event) )) = &beta;_0 + &beta;_1 X_1 + &beta;_2 X_2 + &beta;_3 X_3 + &beta;_4 X_4 + ,&hellip;, + &beta;_k X_k$

It can be summarized as follows: $ln(P(Y&le;j|x)/(1-P(Y&le;j|x) ))=&alpha;_j+&beta;_i X_k$, where $&alpha;_j$ = threshold or intercept, $&beta;_i$ = parameter in the model, and *X *= set of factors or independent variables.

The equation $ln(P(Y&le;j|x)/(1-P(Y&le;j|x) ))=&alpha;_j+&beta;_i X_k$ is an ordinal logistic model for k predictors with the p-1 level response variable [17].

Multilayer Feed-Forward Neural Network (MLFFNN)

The study will employ the multilayer feed-forward neural network (MLFFNN) procedure, which is a widely used artificial neural network. MLFFNN typically consists of three primary layers: the input layer, the hidden layer, and the output layer [18]. In this research, since there is only one dependent variable, the output node for this analysis remains fixed at one. The following equation illustrates the configuration of the MLFFNN model, featuring N input nodes, H hidden nodes, and one output node. The values are given as follows: $ŷ=g_i (\sum _{j=1}^{H} w_j h_j+w_0)$, where wj is an output weight from hidden node j to the output node, the output node bias, and g is an activation function. The values associated with the hidden node h_j_, j =1…H are as follows: $h_j=g_i (\sum _{j=1}^{H} v_{ji}x_{i} + v_{j0})$, where *v_ji_* is the output weight from input node i to hidden node j; *V_j0_* is the bias for hidden node j where j =1, …, H; *x_i_* are the independent variables where i =1, …, N; and *k* is an activation function [19].

**Figure 1:**
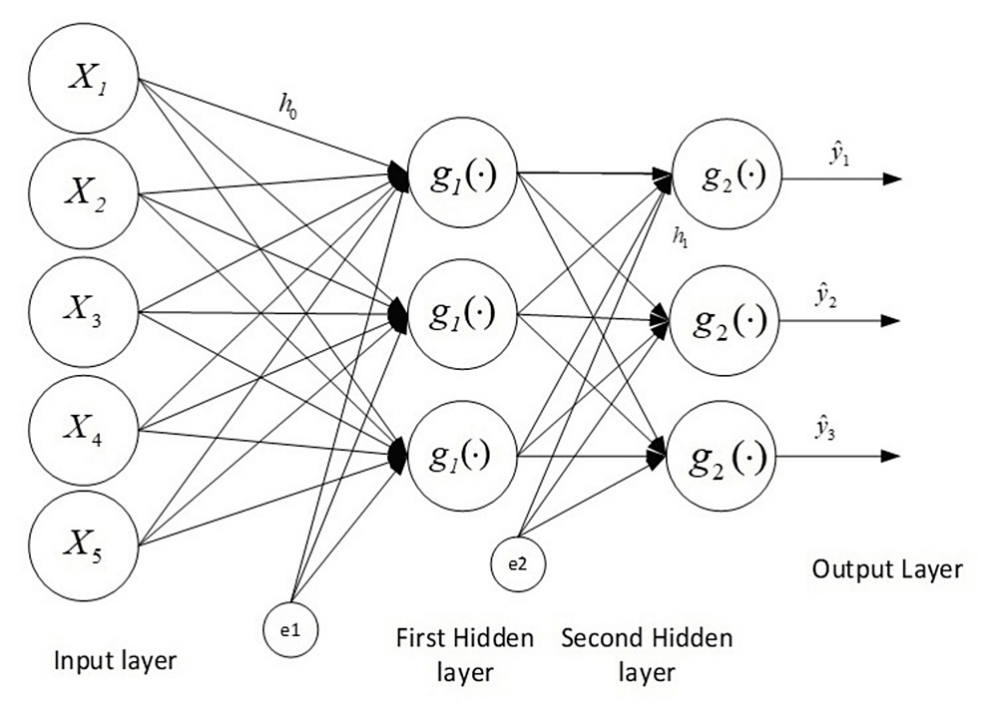
The general architecture of the MLFFNN with one hidden layer, N input nodes, two hidden nodes, and three output nodes MLFFNN: multilayer feed-forward neural network

The general architecture of the MLFFNN model is illustrated in Figure 1.

The Hybrid Method

**Figure 2:**
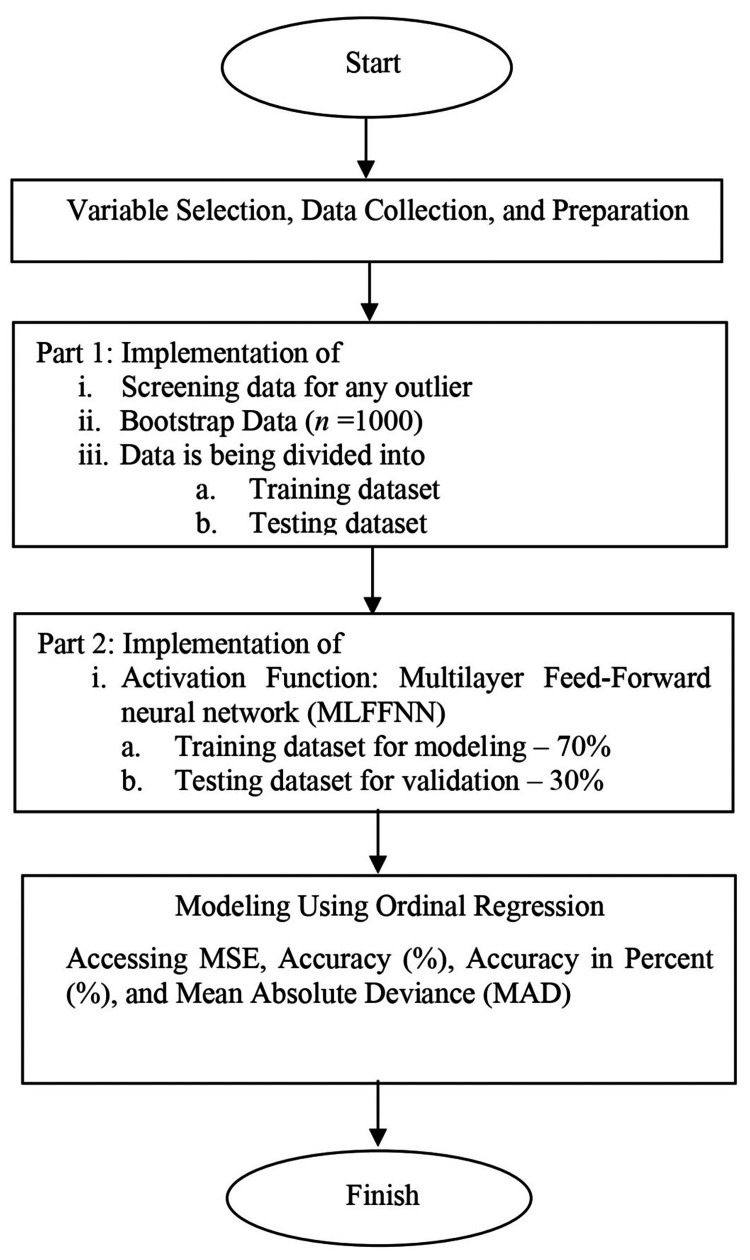
Flowchart of the proposed statistical ordinal modeling MSE: mean square error

The complete step-by-step of the proposed hybrid method is given in Figure 2.

Figure 2 is a diagram showing how this process works. The process of variable selection, data collection, and data preparation is crucial in this study. Variable selection involves identifying and choosing the relevant independent variables that have an impact on the dependent variable. This ensures that the model captures the most important factors influencing the outcome. After the data has been prepared, a bootstrapping method will be constructed. The bootstrap technique involves generating a new sample of the same size as the original by repeatedly selecting observations from the original sample, allowing for the possibility of selecting the same observation multiple times. The observations that are not selected in the bootstrap sample are discarded.

## Results

The primary objective of this study is to assess the effectiveness of a multilayer feed-forward neural network (MLFFNN) utilizing the ordered logistic model as its activation function. Both the training and testing datasets are taken into consideration in this evaluation. The MLFFNN algorithm is employed to identify the optimal model for ordered logistic regression by selecting clinically significant variables that can minimize the predicted mean square error (PMSE).

The result of MLFFN modeling

**Figure 3:**
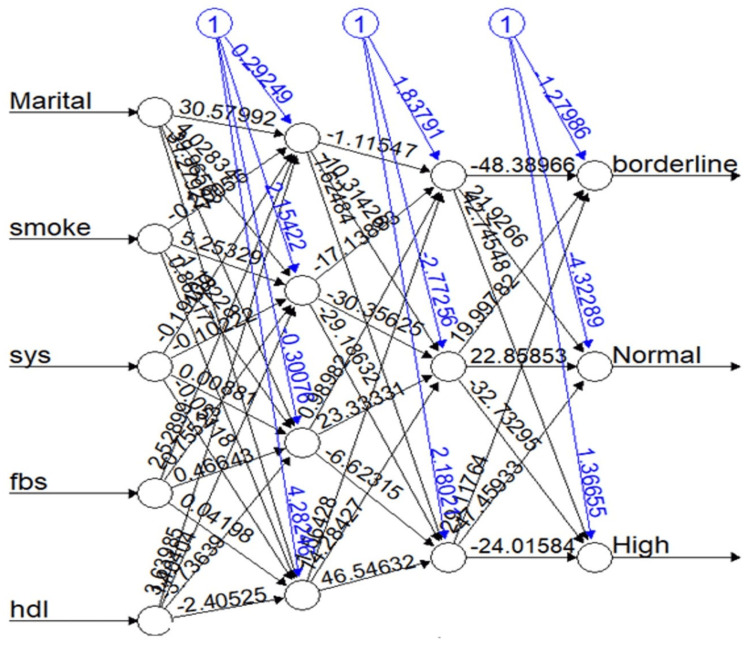
The architecture of the best MLFFNN model with five input variables, two hidden layers, and three output nodes MLFFNN: multilayer feed-forward neural network

Figure 3 shows the network architecture of the best MLFFNN model performed on the training dataset, where hypertension status serves as the dependent variable and the main focus of the study. The image, generated by running the MLFFNN model in RStudio, illustrates the specific model tailored to the supplied dataset. The mean absolute deviance (MAD) value of 0.2614893 indicates the degree to which the available data is accurately distributed. A lower value suggests a more effective analysis, indicating a closer match between the predicted and actual data and a smaller variance. On the other hand, a larger variance indicates significant differences between dissimilar data points. In this study, a 70:30 train-to-test split was employed, meaning that 70% of the data was used for modeling and 30% for testing. This allows us to demonstrate the accuracy and reliability of our predicted data.

In this section (Table 2), the established bootstrap method was utilized to validate the selected factors (variables) in the integrated ordered logistic regression. Five variables were chosen for analysis in this case: marital status (β1, -17.12343; p < 0.25), smoking status (β2, 1.860691; p < 0.25), systolic reading (β3, 0.050373; p < 0.25), fasting blood sugar (β4, -0.538803; p < 0.25), and high-density lipoprotein (β3, 5.380655; p < 0.25). These five factors were found to have a significant effect on hypertension. Table 2 summarizes the detailed output.

**Table 2:** Result of ordered logistic regression by combining the bootstrap method training and testing dataset Ordered logistic regression was applied *Significant at the level of 0.25

Variable	Estimate	Standard Error	Z-value	P-value
Marital Status	-17.12343	0.000016	0.000001	0.000000*
Smoking Status	1.860691	0.919605	2.023359	0.043036*
Systolic Reading	0.050373	0.037780	1.333316	0.182428*
Fasting Blood Sugar	-0.538803	0.198959	-2.708113	0.006766*
High-Density Lipoprotein	5.380655	2.658547	2.023909	0.042979*

Evaluation of the model

In this case, the forecast value can be used to calculate the model evaluation. A tabulation of the actual and predicted values will reveal the prediction's accuracy. The training dataset will be utilized to evaluate the proposed model constructed from the dataset as shown in Table 3.

**Table 3:** Confusion matrix and statistics Accuracy, 88.24%; 95% CI, 0.7255-0.967

	Reference			
Prediction	Prediction	Borderline	High	Normal
	Borderline	11	0	4
	High	0	8	0
	Normal	0	0	11
Statistics by Class		Class: Borderline	Class: High	Class: Normal
	Sensitivity	1.0000	1.0000	0.7333
	Specificity	0.8261	1.0000	1.0000
	Positive Predictive Value	0.7333	1.0000	1.0000
	Negative Predictive Value	1.0000	1.0000	0.8261
	Balanced Accuracy	0.9130	1.0000	0.8667

The proposed MLFFNN-based model achieved a validation accuracy of 99.82%, while the accuracy of the predictions was 88.24%. The proposed methodology demonstrates good performance in terms of sensitivity, specificity, and positive predictive value. Sensitivity and specificity analysis is crucial for evaluating the effectiveness of a test. High specificity indicates accurate analysis, while high sensitivity signifies the test's ability to correctly identify positive cases.

## Discussion

Our successful implementation of the proposed method has proved to be highly valuable for estimating event probabilities, specifically in predicting the likelihood of being a case. By employing a hybrid approach, we were able to develop a model that is both highly accurate and reliable. Traditional regression modeling comes with limitations, particularly in the estimation process where the calculation procedures for predictor variables and outcomes tend to be more complex, less accurate, and less precise. However, our proposed method, which utilizes a single syntax calculation, addresses these limitations and significantly improves the accuracy and precision of ordered regression modeling. The results indicate that hypertension is primarily influenced by factors such as marital status, smoking status, systolic blood pressure reading, fasting blood glucose, and high-density lipoprotein. These factors were found to be the most significant in determining the presence of hypertension. The variable selection process takes into account clinical expert opinion. It begins by creating a consolidated "mega" file from the initial dataset. The bootstrap procedure is then applied to generate a large sample of file replacements. These replacements are used to generate and store samples of statistical data through the bootstrap method. This iterative process is typically repeated thousands of times to ensure comprehensive results. The integration of the methodology concept with the R syntax algorithm enables the application of the proposed method. In variable selection, the initial step involves consulting a clinical expert. Finally, the dataset undergoes the bootstrap procedure, where different sets of data are utilized for training and testing purposes.

Over the past decade, there has been a surge in research investigating the various risk factors associated with hypertension. A clinical approach with enhanced robustness in identifying these risk factors can be achieved by integrating MLFFNN with ordered regression analysis. For instance, in a study by Chang et al., a different mining tool was employed, revealing significant results that underscored the association of triglycerides, creatinine, age, and uric acid with hypertension risk [20]. Similarly, Akdag et al. utilized decision trees to identify BMI, waist/hip ratio, gender, and triglycerides as notable risk factors for hypertension [21]. Another study conducted in Qatar by AlKaabi et al. yielded comparable results using random forest and logistic regression analyses, emphasizing the importance of age, physical activity, fruit and vegetable consumption, and diabetes history as critical predictors of hypertension [5]. Additionally, a longitudinal study by Dimitriadis et al. established a significant association between hypertension and risk factors such as age, gender, and blood glucose levels [22].

The R syntax algorithm serves as a bridge between the application and the concept of a method-based methodology. The initial step involves variable selection, with guidance and input from a medical expert. Subsequently, the bootstrap procedure is applied to the dataset, creating both training and testing datasets. Currently, the bootstrap data is divided such that 30% is allocated as the testing dataset, while the remaining 70% is categorized as the training dataset. The success of a model is determined by its ability to minimize the mean absolute deviation. The calculation of this formula using the provided syntax, based on actual and predicted values, helped in obtaining valuable findings that aided decision-making for achieving optimal outcomes. By incorporating statistical formulations, utilizing R syntax for computation, and employing the ordered logistic regression package, the ordered modeling approach yielded highly successful results. Selecting appropriate input parameters, preparing the data for ordered logistic modeling, and standardizing it are identified as the most challenging tasks in this process.

It is important to acknowledge certain limitations of our study. One notable limitation lies in the generalizability of the findings, as the model was developed based on a specific dataset and may exhibit variations in performance when applied to different populations or settings. The bootstrap procedure, while enhancing robustness, is subject to potential sampling variations and may not account for all possible data scenarios. The challenges in machine learning, such as overfitting or selection bias, could also impact the performance and interpretation of the model. It is crucial for future research to explore the external validity of the model across diverse populations and consider alternative methodologies that address the identified limitations for a more comprehensive understanding of hypertension risk factors.

## Conclusions

This groundbreaking study represents a significant leap forward in the field of hypertension risk factor analysis, aiming to pioneer novel hybrid methods that seamlessly integrate bootstrapping, multilayer neural networks, and ordered logistic regression. The meticulous development of the R syntax, serving as the implementation vehicle for this innovative methodology, ensures clarity and accessibility in its application. The true efficacy of our hybrid model comes to light through its predictive power, with the smallest error obtained serving as a robust indicator of its test fit. By leveraging this hybrid approach, we gain profound insights into its effectiveness and discern its unique contribution to outcomes, elevating our understanding of hypertension risk factors to new heights. The statistical analyses presented in this R study affirm the supremacy of regression modeling, evidenced by an impressively low mean absolute deviation error of 0.2615. These findings underscore the exceptional performance of our proposed hybrid model technique, firmly positioning it as a front-runner in the realm of hypertension risk assessment and surpassing alternative methods. As we forge ahead, these results not only validate the superiority of our approach but also pave the way for future advancements in predictive modeling, heralding a new era in the quest for precision and accuracy in hypertension research.
